# Melissa officinalis L. Supplementation Provides Cardioprotection in a Rat Model of Experimental Autoimmune Myocarditis

**DOI:** 10.1155/2022/1344946

**Published:** 2022-02-28

**Authors:** Nevena D. Draginic, Vladimir L. Jakovljevic, Jovana N. Jeremic, Ivan M. Srejovic, Marijana M. Andjic, Marina R. Rankovic, Jasmina Z. Sretenovic, Vladimir I. Zivkovic, Biljana T. Ljujic, Slobodanka L. Mitrovic, Stefani S. Bolevich, Sergey B. Bolevich, Isidora M. Milosavljevic

**Affiliations:** ^1^Department of Pharmacy, Faculty of Medical Sciences, University of Kragujevac, 34000 Kragujevac, Serbia; ^2^Department of Human Pathology, First Moscow State Medical University I.M. Sechenov, Moscow, Russia; ^3^Department of Physiology, Faculty of Medical Sciences, University of Kragujevac, 34000 Kragujevac, Serbia; ^4^Department of Genetics, Faculty of Medical Sciences, University of Kragujevac, 34000 Kragujevac, Serbia; ^5^Department of Pathology, Faculty of Medical Sciences, University of Kragujevac, 34000 Kragujevac, Serbia; ^6^Department of Pathophysiology, 1st Moscow State Medical University IM Sechenov, Moscow, Russia

## Abstract

Due to existing evidence regarding antioxidant and anti-inflammatory effects of *Melissa officinalis extracts* (MOEs), this study was aimed at investigating the potential of ethanolic MOE to prevent the development of myocarditis and its ability to ameliorate the severity of experimental autoimmune myocarditis (EAM) by investigating MOE effects on *in vivo* cardiac function, structure, morphology, and oxidative stress parameters. A total of 50 7-week-old male *Dark Agouti* rats were enrolled in the study and randomly allocated into the following groups: CTRL, nontreated healthy rats; EAM, nontreated rats with EAM; MOE50, MOE100, and MOE200, rats with EAM treated with either 50, 100, or 200 mg/kg of MOE for 3 weeks *per os*. Myocarditis was induced by immunization of the rats with porcine myocardial myosin (0.5 mg) emulsion on day 0. Cardiac function and dimensions of the left ventricle (LV) were assessed via echocardiography. Additionally, the blood pressure and heart rate were measured. On day 21, rats were sacrificed and the hearts were isolated for further histopathological analyses (H/E and Picrosirius red staining). The blood samples were collected to determine oxidative stress parameters. The EAM group characteristically showed greater LV wall thickness and lower ejection fraction (50.33 ± 7.94% vs. 84.81 ± 7.74%) and fractional shortening compared to CTRL (*p* < 0.05). MOE significantly improved echocardiographic parameters (EF in MOE200 81.44 ± 5.51%) and also reduced inflammatory infiltrate (by 88.46%; *p* < 0.001) and collagen content (by 76.39%; *p* < 0.001) in the heart tissues, especially in the MOE200 group compared to the EAM group. In addition, MOEs induced a significant decrease of prooxidants production (O_2_^−^, H_2_O_2_, and TBARS) and improved antioxidant defense system via increase in GSH, SOD, and CAT compared to EAM, with medium and high dose being more effective than low dose (*p* < 0.05). The present study suggests that ethanolic MOEs, especially in a 200 mg/kg dose, improve cardiac function and myocardial architecture, possibly via oxidative stress mitigation, thus preventing heart remodeling, development of dilated cardiomyopathy, and subsequent heart failure connected with EAM. MOEs might be considered as a potentially helpful adjuvant therapy in patients with autoimmune myocarditis.

## 1. Introduction

Myocarditis is an inflammatory heart disease characterized by nonischemic inflammatory infiltrates in the heart tissue associated with necrosis and/or degeneration of cardiomyocytes. Several entities have been identified as the causes of myocarditis, such as viral or bacterial infections, drugs or toxin usage, and autoimmune processes. Acute myocardial inflammation may progress to subacute and chronic phases and ultimately lead to tissue remodeling, fibrosis, myocardium architecture damage, and depressed contractile function [1]. Autoimmune myocarditis, also known as *giant cell* myocarditis, is associated with poor prognosis since it often leads to dilated cardiomyopathy (DCM) in chronic stages. It is also estimated that one-third of autoimmune myocarditis cases develop heart failure, and almost 40% of all heart failures in the population under 40 is actually associated with autoimmune myocarditis. In addition, autoimmune myocarditis tends to be unrecognized in patients with DCM, until *post mortem* histopathological analyses, as its clinical presentation varies widely [2].

Taking into account that current therapeutic options for myocarditis are limited to symptomatic treatment for arrhythmias and heart failure and that no effective therapeutic strategy has been developed yet [2], the search for novel efficient therapeutic options is necessary. The most commonly used animal model of *giant cell* autoimmune myocarditis is EAM on rodents, which allows investigation of the mechanisms involved in this pathology, as well as testing of novel treatments [2–4]. The usage of natural products in the treatment of cardiovascular disease is gaining popularity owing to their safety, fewer side effects, and lower costs. In recent years, preclinical and clinical research has been focused on identifying innovative phytomedicines, including plant extracts with high anti-inflammatory and antioxidant potential, and especially on identifying active components responsible for cardioprotection [5, 6].

*Melissa officinalis* L. (*Lamiaceae*), also known as lemon balm, is a perennial herb belonging to lemon scent, mint family of plants. It has been widely used in traditional medicine for centuries, mainly because of its beneficial effects on the nervous system, including anxiety symptoms and palpitation relieving, mild sedative, and hypnotic effects. In addition, a plethora of pharmacological activities of lemon balm have been described: hypoglycemic, hepatoprotective, antibacterial, anti-inflammatory, antioxidant, antiviral, antispasmodic, neuroprotective, and cytotoxic effects. Literature data also suggests its beneficial effects on the cardiovascular system such as antiarrhythmic and vasorelaxant properties and protective effects in myocardial ischemia-reperfusion injury [7–9]. These cardiovascular effects of MOEs are connected to their antioxidant potential and free radical scavenging properties. Polyphenolic compounds particularly rosmarinic acid, as the major component, but also cinnamic, protocatechuic, caffeic, ferulic, and ellagic acids; flavonoids (quercetin, luteolin, apigenin, catechin, epicatechin, and rutin); and triterpenoids ursolic and oleanolic acids are highlighted as the active compounds responsible for MOE antioxidant potential. Additionally, anti-inflammatory potential of MOEs has been proven in the carrageenan-induced paw edema model, which may be very useful in EAM [10, 11]. It is suggested that modulation of immune response by MOEs is achieved by strong anti-inflammatory potential of rosmarinic acid and triterpenoids.

Up until now, the beneficial effects of bioactive plant compounds such as curcumin, quercetin, apigenin, berberine, resveratrol, oleanolic acid, catechin, and epigallocatechin have been confirmed in EAM model [12–14]. Several mechanisms mediate these beneficial effects, including modulation of oxidative stress, suppression of apoptosis and fibrosis, and modulation of the immune response and cytokine concentration [5, 6, 15–17]. Nonetheless, the effects of MOEs in this pathology are entirely unexplored.

Considering proven antioxidant and anti-inflammatory effects of MOEs, we aimed to investigate the potential of ethanolic MOE to prevent the development of myocarditis and its ability to ameliorate the severity of EAM by investigating MOEs effects on *in vivo* cardiac function, structure, morphology, and oxidative stress parameters.

## 2. Materials and Methods

### 2.1. Ethical Standards

All experimental procedures involving laboratory animals used in this research were approved by Ethics Committee for experimental animal well-being of the Faculty of Medical Sciences, University of Kragujevac (Kragujevac, Serbia) No. 01-10171. Furthermore, all the experimental procedures were performed according to European Directive 2010/63/EU for the welfare of laboratory animals, number and principles of Good Laboratory Practice (GLP) (86/609/EEC). Additionally, experiments were carried out following the European Union Directive 86/609/EES for the Protection of the Vertebrate Animals used for Experimental and other Scientific Purposes and the principles of ethics.

### 2.2. Plant Material and Plant Extraction

For the purposes of this research, dried leaves of *Melissa officinalis* L. (*Lamiaceae*) purchased from *Bilje Borca*, *LLC* (*Belgrade*, *Serbia*) were used. The dried plant material was pulverized with a mill (*IKA A11*, *Germany*) and stored in well-sealed paper bags at room temperature until the extract was made. The ethanolic MOE was obtained under the reflux of the solvent. This method involves extraction at the boiling point of the solvent (70% ethanol). The extraction was performed for 2.5 hours, after which the mixture was filtered through gauze and left at room temperature to spontaneously precipitate ballast substances. Finally, the obtained liquid extract was filtered (*Whatman*, *No. 1*), while we used a rotary vacuum evaporator (*RV05 basic IKA*, *Germany*) at 40°C, 90 rpm, and 250 mbar vacuum to obtain dry extract, which was stored in dark glass vials at +4°C until administration [18].

### 2.3. Animals

The study involved a total of 50 seven-week-old male *Dark Agouti* (DA) rats, weighing 150 ± 20 g at the beginning of the experiment, purchased from the Military Medical Academy Animal House, Belgrade. Firstly, animals were acclimatized for two weeks and kept in polyethylene cages (4 per cage) under standardized controlled environmental conditions (22 ± 2°C and a 12 h light/dark cycle). Free access to standard food (9% fat, 20% protein, and 53% starch) and water (*ad libitum*) was provided for all animals.

### 2.4. Induction of Experimental Autoimmune Myocarditis

Calcium-activated myosin from the porcine heart (*Sigma-Aldrich*, *Munich*, *Germany*) was dissolved in 0.01 M phosphate-buffered saline (PBS) in one tube and emulsified with an equal volume of complete Freund's adjuvant (FCA) supplemented with Mycobacterium tuberculosis (*strain H37 RA; Difco Laboratories*, *Detroit*, *MI*) at a concentration of 10 mg/ml mixed in a separate tube. The suspensions from both tubes were then mixed, vortexed, and transferred to a syringe. The suspension was then homogenized by moving the content back and forth between the two syringes for 60 min. The final volume of the suspension was drawn into a 1 ml sterile syringe with Luer-Lock tip and connected to a 26G needle. The suspension was prepared *ex tempore* on the day of immunization. On day 0, the rats were injected subcutaneously into both rear hind footpads with 0.1 ml of final emulsion (0.05 ml per footpad), yielding an immunizing dose of 0.25 mg/body of cardiac myosin per rat. The CFA emulsified with PBS was applied to the control group [19].

### 2.5. Study Design

The rats (*n* = 50) were randomly allocated into five different groups: CTRL, healthy nontreated rats; EAM, nontreated rats with myocarditis; and MOE50, MOE100, and MOE200, rats with myocarditis treated with three different doses (50 mg/kg, 100 mg/kg, and 200 mg/kg) of ethanolic MOEs. Treatment involved daily *per os* application (every day at the same time) of MOE dissolved in distilled water, *ex tempore* (volume of 300 *μ*l approximately). All animals were weighed during the protocol to adjust the MOE dose according to the rats' body weight.

### 2.6. Hw/Bw and Sw/Bw Ratios

The rat's body weight was measured directly before *in vivo* functional analysis. Afterward, the rats were sacrificed and the hearts and spleens were isolated and measured in order to calculate relative heart weight (Hw) and spleen weight (Sw) to body weight (Hw/Bw and Sw/Bw) ratios.

### 2.7. Blood Pressure and Heart Rate Measurement

The systolic and diastolic blood pressures (SBP and DBP) and heart rate (HR) were measured by a tail-cuff noninvasive method BP system (*Rat Tail Cuff Method Blood Pressure Systems (MRBP-R)*, *IITC Life Science Inc.*, *Los Angeles*, *CA*, *USA*) twice, first at the beginning of the experimental protocol (day 0) in order to check the homogeneity of the animals, when no difference was found, and then after accomplishing the 3-week protocol before sacrificing the animals (day 21) [20].

### 2.8. Echocardiographic Analyses

Transthoracic echocardiography was performed to assess the effects of MOE on *in vivo* cardiac function and the development of autoimmune myocarditis. The procedure was repeated twice, first at the beginning (day 0) to check the homogeneity of the animals and their health, when no difference was found, and then at the end of the experimental protocol (day 21) before sacrificing the animals. The animals were anesthetized with mixture of ketamine (75 mg/kg) and xylazine (5 mg/kg) intraperitoneally. Echocardiograms were performed using a *Hewlett-Packard Sonos 5500 (Andover*, *MA*, *USA)* sector scanner equipped with a 15.0 MHz phased-array transducer as in our previous research [20]. From the parasternal long-axis view in 2-dimensional mode, and M-mode cursor was positioned perpendicularly to the interventricular septum and posterior wall of the left ventricle (LV) at the papillary muscle level and M-mode images were obtained. The following parameters were measured: interventricular septal wall thickness at end-diastole (IVSd), LV internal dimension at end-diastole (LVIDd), LV posterior wall thickness at end-diastole (LVPWd), interventricular septal wall thickness at end-systole (IVSs), LV internal diameter at end-systole (LVIDs), and LV posterior wall thickness at end-systole (LVPWs) were recorded with M-mode. Fractional shortening percentage (FS%) was calculated from the M-mode LV diameters using the equation [(LVIDd − LVIDs)/LVIDd] × 100%, where LVIDd is left ventricular end diastolic diameter and LVIDs is left ventricular end-systolic diameter. Ejection fraction (EF%) was calculated according to the *Teichholz* formula [21], where LVEDV represents LV end-diastolic volume, while LVESV represents LV end-systolic volume.

(1) $$\begin{array}{c} EF=100\times \frac{\left(LVEDV-LVESV\right)}{LVEDV}LVESV=\frac{\left(7\times LVIDs\right)}{\left(2.4\times LVIDs\right)}LVEDV=\frac{\left(7\times LVIDd\right)}{\left(2.4\times LVIDd\right)} \end{array}$$

### 2.9. Biochemical Analyses-Oxidative Stress Parameters

After completing the 3-week protocol, all animals were anesthetized by short ketamine and xylazine narcosis and sacrificed by decapitation. The blood samples were collected to determine oxidative stress parameters spectrophotometrically (*Shimadzu UV 1800 spectrophotometer*, *Kyoto*, *Japan*). The blood samples were centrifuged in order to separate the plasma and obtain red blood cell (RBC) lysate suspension by washing isolated separated RBCs 3 times with ice cold saline. The following prooxidant parameters were determined from plasma samples: superoxide anion radical (O_2_^−^), hydrogen peroxide (H_2_O_2_), nitrites (NO_2_^−^), and index of lipid peroxidation measured as thiobarbituric acid reactive substances (TBARS). Antioxidant protection parameters were determined from erythrocyte lysate samples: the activity of catalase (CAT) and superoxide-dismutase (SOD) and the level of reduced glutathione (GSH).

#### 2.9.1. Determination of Prooxidants (O_2_^−^, H_2_O_2_, NO_2_^−^, and TBARS)

The quantification of superoxide anion radical was based on the reaction of O_2_^−^ with nitro blue tetrazolium (NBT). The protocol included mixing of 50 *μ*l of plasma samples and 950 *μ*l of assay mixture, followed by measuring on 550 nm in triplicate every 60 s [22].

The hydrogen peroxide (H_2_O_2_) determination method was based on the oxidation of phenol red with horseradish peroxidase enzyme. 200 *μ*l of plasma sample was mixed with 800 *μ*l of PRS (phenol red solution) and 10 *μ*l POD (horseradish peroxidase (1 : 20)). Measuring was performed at 610 nm [22].

Nitric oxide (NO) level was assessed indirectly by measuring nitrite concentration, since NO decomposes rapidly forming an equal amount of nitrite products. First, 100 *μ*l of PCA (perchloride acid), 400 *μ*l of 20 mM ethylenediaminetetraacetic acid (EDTA), and 200 *μ*l of the plasma sample were mixed, put on the ice for 15 min, and centrifuged for 15 min at 6000 rpm. After separating the supernatant, 220 *μ*l K_2_CO_3_ was added. Measuring was performed at 550 nm [22].

Index of lipid peroxidation in the plasma samples was estimated indirectly by measuring TBARS. First, TBA extract was made by mixing 800 *μ*l sample and 400 *μ*l trichloroacetic acid (TCA), which was then put on ice for 10 min and centrifuged for 15 min at 6000 rpm. Next, 1% TBA (thiobarbituric acid) in 0.05 NaOH was incubated with the obtained sample at 100°C for 15 min and after 10 min measured at wavelength of 530 nm [22].

#### 2.9.2. Determination of Antioxidants (SOD, CAT, and GSH)

Obtained lysates containing about 50 g Hb/l were used to determine antioxidant enzyme activity. CAT buffer, sample, and 10 mM H_2_O_2_ were used for CAT determination. Detection was performed at 360 nm [22]. SOD activity was evaluated by the epinephrine method. Lysate sample was first mixed with carbonate buffer, and then epinephrine was added. Detection was performed at 470 nm. The amount of SOD and CAT was expressed as U/g Hb ×103 [23, 24]. The reduced glutathione (GSH) level was determined by GSH oxidation with 5,5-dithiobis-6,2-nitrobenzoic acid. GSH extract was made by mixing 100 *μ*l 0.1% EDTA, 400 *μ*l lysate, and 750 *μ*l precipitation solution (1.67 g metaphosphoric acid, 0.2 g EDTA, 30 g NaCl, and filled with distilled water to 100 ml). This was followed by mixing in the vortex machine and extraction on cold ice (15 min) and centrifugation at 4000 rpm (10 min). Distilled water was used as a blank probe. The level of GSH was measured at 420 nm and expressed as nanomoles per milliliter of RBCs [22].

### 2.10. Histological Analyses of the Heart

The isolated hearts were measured and then cut into two halves so that the left and right halves of the heart were available for further histological analysis. The hearts were then fixed in 4% neutral paraformaldehyde, dehydrated in increasing alcohol concentrations (70%, 96%, and 100%), cleared in xylene, immersed in paraffin, and prepared for further analysis. 5 *μ*m thick serial sections were stained by the H/E (hematoxylin/eosin) method for the purpose of morphometric analysis of cells and verification of morphological changes and by the Picrosirius red staining for collagen detection. Images of heart tissue sections were taken on an *Olympus BX51 light microscope*. Morphometric analysis of cardiomyocytes (longitudinal section diameter as well as cross-sectional area) was performed in the *Axiovision image analysis program* (*Zeiss*, *USA*), where 100-120 cells per animal were analyzed [25]. The cell infiltrate density and the collagen content were analyzed using *Image Pro-Plus programs (Media Cybernetics*, *USA)*. The analysis of cell infiltrate density and the collagen content was performed on 10 sections, of the total number of serial sections of the heart, with every 20^th^ section of heart tissue analyzed, i.e., the distance between the analyzed plates was 100 *μ*m. The results are presented as percentages. It is important to emphasize that no cell infiltrate was verified in the control group, and the value for infiltrate density was presented as 0%.

### 2.11. Statistical Analyses

*IBM SPSS 20.0* was used for statistical data processing for Windows. The Kolmogorov-Smirnov and Shapiro-Wilk tests, histogram, and normal QQ plot tests were used to examine the normality of the distribution. Data are expressed as mean value (X) ± standard deviation (SD) and analyzed by one-way analysis of variance (ANOVA), followed by the Bonferroni test. A value of *p* < 0.05 was considered significant.

## 3. Results

### 3.1. Effects of MOE on Hw/Bw and Sw/Bw Ratios

Immunized nontreated EAM rats and rats treated with a low dose of MOE (MOE50) were shown to have significantly increased heart weights and Hw/Bw ratio compared to the healthy CTRL group (*p* < 0.01). Additionally, medium and high doses of MOE (groups MOE100 and MOE200) significantly lowered heart weights and Hw/Bw ratio compared to both EAM and MOE50 groups (*p* < 0.05). The EAM group also showed a significantly increased Sw and Sw/Bw ratio compared to CTRL, while MOE100 and MOE200 significantly lowered these two parameters. The Sw/Bw ratio reduction was the most prominent in the MOE200 group (Table 1*).*

### 3.2. Effects of MOE on Hemodynamic Parameters

Three weeks post-immunization, significantly elevated HR was observed in the EAM group compared to CTRL, while treatment with extract induced a significant HR reduction in all three dose regimens compared to EAM. Additionally, medium and high doses of extract in combination with EAM induced a significant HR reduction compared to CTRL. Systolic blood pressure was shown to be lowered in the MOE200 group compared to the CTRL, EAM, and MOE50 groups (*p* < 0.05), while no significant differences in this parameter were noticed between other groups. Also, no significant changes in DBP were observed between groups (Table 2).

**Figure 1:**
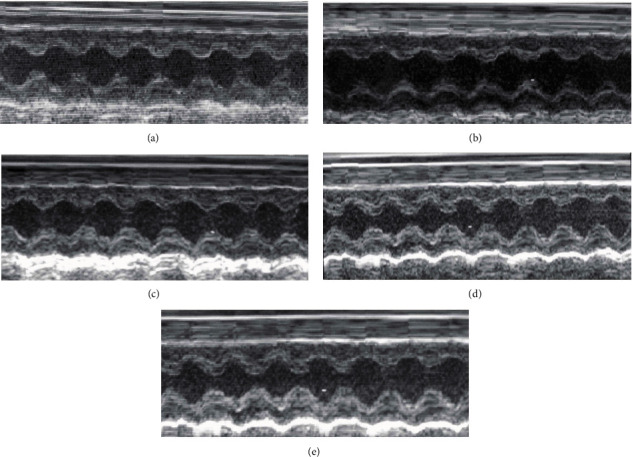
Representative M-mode echocardiograms. (a) CTRL, (b) EAM, (c) MOE50, (d) MOE100, and (e) MOE200. CTRL: control group; EAM: rats with experimental autoimmune myocarditis; MOE50, MOE100, and MOE200: groups of rats treated with either 50, 100, or 200 mg/kg of *M. officinalis* extract.

Significantly decreased ejection fraction was observed in immunized EAM and MOE50 rats compared to CTRL animals (50.33% and 53.89% vs. 84.82%, *p* < 0.01), while treatment with MOE100 and MOE200 (72.47% and 81.44%) markedly improved EF after 3-week supplementation compared to both the EAM and MOE50 groups. Only the highest dose MOE200 succeeded in normalizing EF to levels similar to CTRL values. A similar trend was observed in the FS parameter, which was significantly lower in EAM rats relative to CTRL, while all three doses of MOE led to an increment of FS compared to the EAM group (*p* < 0.05). The EAM group was also associated with LVPWd and LVPWs thickening compared to healthy rat hearts, all three doses of MOE led to reduction of these parameters, while MOE200 seemed to normalize these values to levels similar to control ones (Figure 1 and Table 3).

### 3.3. Effects of MOE on Oxidative Stress Parameters

**Figure 2:**
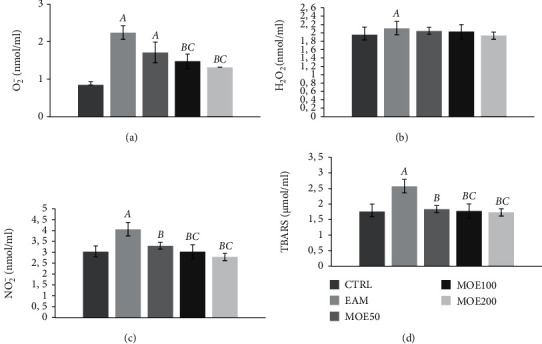
Effects of MOE on prooxidant parameters. (a) Superoxide anion radical (O_2_^−^), (b) hydrogen peroxide (H_2_O_2_), (c) nitrites (NO_2_^−^), and (d) index of lipid peroxidation measured as thiobarbituric acid reactive substances (TBARS). CTRL: control group; EAM: rats with experimental autoimmune myocarditis; MOE50, MOE100, and MOE200: groups of rats treated with either 50, 100, or 200 mg/kg of *Melissa officinalis* extract. Data are presented as means ± standard deviation. Statistical significance at the level *p* < 0.05: A, compared to CTRL; B, compared to EAM; C, compared to MOE50; D, compared to MOE100; and E, compared to MOE200.

**Figure 3:**
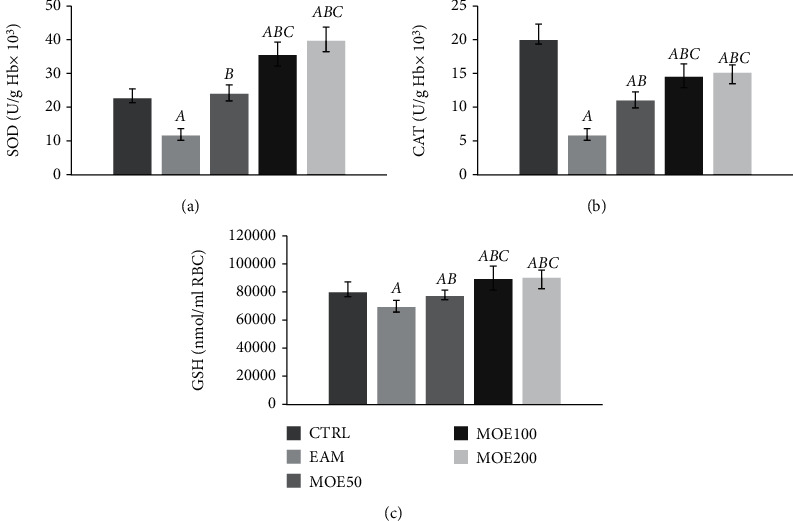
Effects of MOE on antioxidant parameters. (a) Superoxide dismutase (SOD), (b) catalase (CAT), and (c) reduced glutathione (GSH). CTRL: control group; EAM: rats with experimental autoimmune myocarditis; MOE50, MOE100, and MOE200: groups of rats treated with either 50, 100, or 200 mg/kg of *Melissa officinalis* extract. Data are presented as means ± standard deviation. Statistical significance at the level *p* < 0.05: A, compared to CTRL; B, compared to EAM; C, compared to MOE50; D, compared to MOE100; and E, compared to MOE200.

Three weeks post-induction of EAM, significantly higher release of all measured prooxidant markers (O_2_^−^, H_2_O_2_, NO_2_^−^, and TBARS) was observed in EAM rats compared to the CTRL group (*p* < 0.05). However, treatment with MOE in all three doses succeeded in significantly decreasing the level of TBARS and NO_2_^−^ (*p* < 0.05) compared to EAM, with no effect on the level of hydrogen peroxide. The level of superoxide anion radical was significantly lowered only by medium and high dose MOE100 and MOE200 compared to EAM. In addition, the highest and medium dose of the applied extract showed a more dominant effect on O_2_^−^, NO_2_^−^, and TBARS compared to MOE50 (Figure 2). Regarding antioxidants, significantly lower activity of antioxidant enzymes CAT and SOD and the level of GSH were observed in the EAM group (*p* < 0.05), while MOE treatment improved antioxidant protection via an increase in all three parameters compared to EAM (*p* < 0.05). Medium and high doses of MOE significantly improved all three parameters compared to EAM and MOE50. Additionally, medium and high dose of MOE significantly improved SOD and GSH compared to the CTRL group (*p* < 0.05). No differences were observed between the MOE200 and MOE100 groups in all 3 measured parameters (*p* > 0.05). Low-dose MOE50 improved SOD and CAT compared to EAM to a lesser extent than MOE100 and MOE200 (Figure 3).

### 3.4. Effects of MOE on Myocardium Structure

#### 3.4.1. Hematoxylin-Eosin Staining

**Figure 4:**
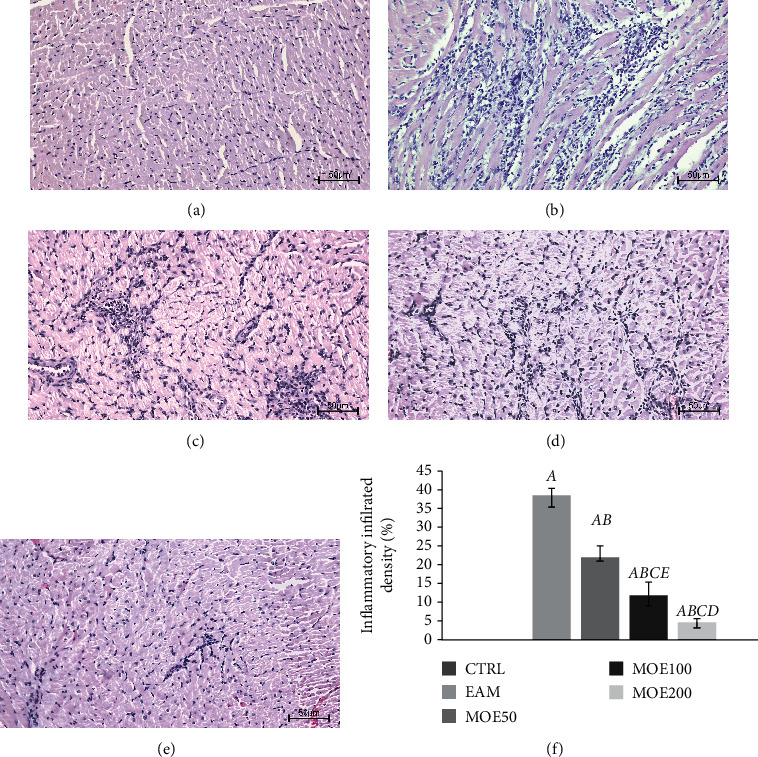
Representative heart tissue sections of H/E staining. Magnification 20x scale bar = 50 *μ*m. (a) CTRL: control group; (b) EAM: experimental autoimmune myocarditis group; (c) MOE50: rats with EAM treated with *M. officinalis* extract in 50 mg/kg; (d) MOE100: rats with EAM treated with *M. officinalis* extract in 100 mg/kg; and (e) MOE200: rats with EAM treated with *M. officinalis* extract in 200 mg/kg. (f) Effects of MOE on heart inflammatory infiltrate density. Data are presented as means ± standard deviation. Statistical significance at the level *p* < 0.05: A, compared to CTRL; B, compared to EAM; C, compared to MOE50; D, compared to MOE100; and E, compared to MOE200.

The preserved myocardial structure was observed in the CTRL group of rats. H-/E-stained sections of the EAM group of rats confirmed the presence of severe myocarditis characterized by massive inflammatory cell infiltration, destruction of myocardial fibers, swelling of cardiomyocytes, interstitial edema, and increased sarcoplasmic eosinophilia. However, MOE treatment especially MOE200 improved cardiac structure after 3-week treatment. EAM heart tissue sections showed a significant presence of inflammatory infiltrate compared to CTRL healthy hearts. Inflammatory infiltrate consisted of different leukocytes including mononuclear cells, polymorphonuclear neutrophils and multinucleated giant cells, which mainly infiltrated the epicardium of the ventricular wall. Namely, all three doses of MOE (MOE50, MOE100, and MOE200) significantly reduced inflammatory infiltrate density (by 44.38%, 71.37%, and 88.46%) compared to the EAM group (Figure 4*).* Additionally, significant differences between different doses of MOE were observed, and a dose-dependent effect was noticed (*p* < 0.01).

#### 3.4.2. Picrosirius Red Staining

**Figure 5:**
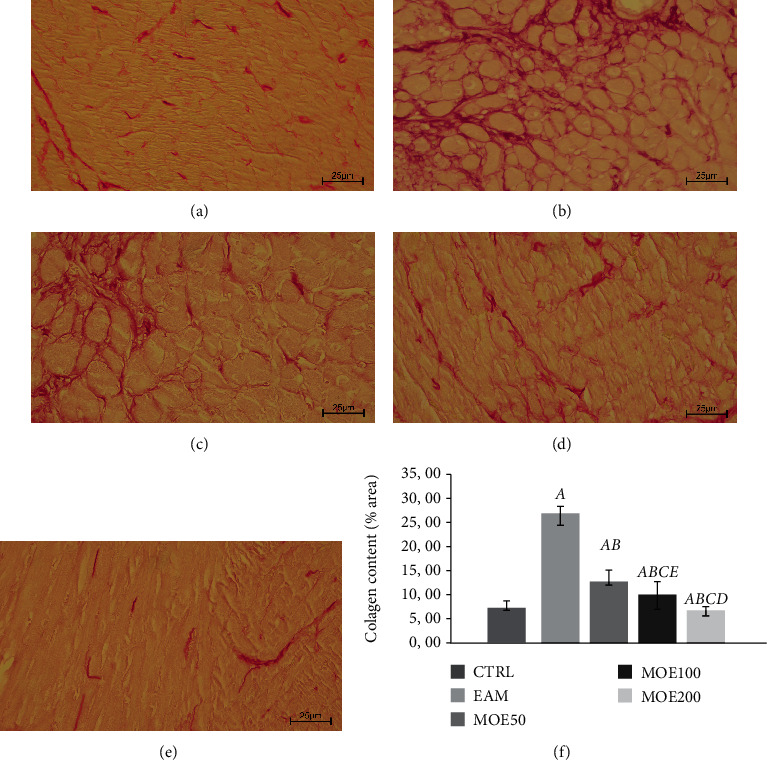
Representative heart tissue sections of Picrosirius red staining. Magnification 40x scale bar = 25 *μ*m. (a) CTRL: control group; (b) EAM: experimental autoimmune myocarditis group; (c) MOE50: rats with EAM treated with *M. officinalis* extract in 50 mg/kg; (d) MOE100: rats with EAM treated with M. officinalis extract in 100 mg/kg; and (e) MOE200: rats with EAM treated with *M. officinalis* extract in 200 mg/kg. (f) Effects of MOE on collagen content in heart tissue. Data are presented as means ± standard deviation. Statistical significance at the level *p* < 0.05: A, compared to CTRL; B, compared to EAM; C, compared to MOE50; D, compared to MOE100; and E, compared to MOE200.

Analysis of Picrosirius red staining in the heart tissue section revealed higher amount of fibrosis in the EAM group, while MOE treatment, especially MOE200, significantly decreased fibrosis compared to EAM, MOE50, and MOE100. Experimental autoimmune myocarditis (EAM group) induced significant almost threefold increase (by 261.31%) in collagen content compared to the CTRL group of healthy rat hearts (*p* < 0.01), while 3-week MOE treatment succeeded to diminish these changes. All three doses of MOE significantly reduced the elevated collagen content compared to EAM rats (MOE50 by 50.23%, MOE100 by 61.39%, and MOE200 by 76.39%) with the most prominent effect noticed in the MOE200 group which normalized the collagen content to CTRL values. Also, a dose-dependent effect of MOE treatment on collagen content was noticed (*p* < 0.05) (Figure 5).

### 3.5. Effects of MOE on Heart Morphometric Parameters

**Figure 6:**
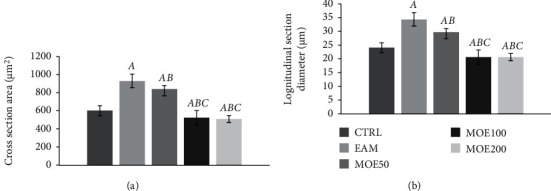
Effects of MOE treatment on morphometric parameters. CTRL: control group; EAM: experimental autoimmune myocarditis group; MOE50, MOE100, and MOE200: rats with EAM treated with *M. officinalis* extract in either 50 mg/kg, 100 mg/kg, or 200 mg/kg. Data are presented as means ± standard deviation. Statistical significance at the level *p* < 0.05: A, compared to CTRL; B, compared to EAM; C, compared to MOE50; D, compared to MOE100; and E, compared to MOE200.

Both cross-section area and longitudinal diameters of cardiomyocytes were significantly elevated in the EAM group of rats compared to the CTRL group (*p* < 0.01). However, MOE treatment significantly decreased these two parameters compared to the EAM group. Medium and high dose of MOE showed superior effects compared to a low dose of MOE (MOE50 group) (*p* < 0.01). No differences in the measured parameters were noticed between MOE100 and MOE200 (Figure 6).

## 4. Discussion

Experimental autoimmune myocarditis in rats is associated with severe changes in the myocardial architecture including massive inflammatory cell infiltration and impaired cardiac function ultimately leading to heart remodeling and dilated cardiomyopathy (DCM). The pathophysiology of this disease is very complex and not fully elucidated yet. However, it is known that excessive ROS production and subsequent oxidative stress induce the release of inflammatory cytokines and chemokines included in leukocytes' migration to the heart tissue. Additionally, oxidative stress may cause cardiomyocyte damage by necrosis or apoptosis [2]. DCM involves the dilatation of the ventricles, which may impair systolic function [1]. Irreversible fiber damage, fibrosis, and finally heart failure may occur as the consequence of systolic dysfunction, leaving the heart transplantation as the only therapeutic option [26]. Even though there is evidence on natural products being useful in EAM pathology [5, 6], to the best of our knowledge, this is currently the first study dealing with the effects of *M. officinalis* in autoimmune myocarditis.

Hemodynamic measurements implicated that the EAM group was associated with a severe drop of ejection fraction (EF) and fractional shortening (FS), LV wall thickening, and increased heart rate. With morphometric changes, heart enlargement by increased Hw/Bw ratio, and cardiomyocyte cross section area and longitudinal diameter increment in EAM rats, all of the above-mentioned indicates disturbed heart function and the beginning of characteristic myocarditis induced DCM. Similar findings are presented in other studies using the EAM model [27, 28]. MOE treatment, especially MOE200, succeeded in improving myocardial function by normalizing EF and FS values and decreasing LVPW and preventing development of left ventricular remodeling and the progression to heart failure following myocarditis. However, recent data suggest that autoimmune myocarditis is not always associated with systolic dysfunction and that there are cases of myocarditis with preserved EF [29]. Furthermore, markedly increased heart rate in EAM was lowered by MOE treatment, which can be ascribed to Melissa's proven ability to act as an antiarrhythmic agent via activation of cardiac M_2_ receptors, blockage of Ca^2+^ and K^+^ channels, and slowing ventricular conductivity [9, 30, 31]. However, we did not observe any changes in the blood pressure of EAM rats unlike others [23]. Nevertheless, MOE200 showed hypotensive effects which can be explained by the previously described vasorelaxant effect of MOE involving Ca^2+^ blockage, nitric oxide pathway, but also prostacyclin and EDHF pathways [15, 24].

In our study, induction of EAM was confirmed histopathologically by characteristic severe inflammatory infiltration and fibrosis of the heart tissues which is in line with other studies using the EAM rat model [14, 27, 32]. Interestingly 3-week treatment with MOEs markedly improved myocardial architecture and decreased inflammatory infiltrate density in a dose-dependent manner, with most pronounced improvement in the MOE200 group. Also, MOE treatment decreased collagen content, suggesting again that MOE200 can prevent myocarditis-induced fibrosis and subsequent heart remodeling [27]. Mentioned effects achieved by MOE administration are most likely connected to its strong anti-inflammatory properties proved *in vivo* in the carrageenan-induced paw edema model [11], but also in other models of cardiovascular diseases [17, 33]. Even though MOEs have not been investigated in EAM pathology, achieved myocarditis ameliorating properties may be associated with synergistic action of its compounds, especially rosmarinic acid and other phenolic acids; triterpenoids oleanolic and ursolic acids; and flavonoids quercetin, rutin, myricetin, catechin, and epigallocatechin. Rosmarinic acid, the most abundant compound of MOEs, is proved to be a very potent anti-inflammatory agent per se, as demonstrated in different models of autoimmune inflammatory disease such as rheumatoid arthritis, colitis, and atopic dermatitis. Possible mechanisms of this action are decreased COX-2 expression and decreased proinflammatory cytokines IL-1, IL-6, and TNF-alpha release [34]. Also, in myocardial I/R injury conditions, rosmarinic acid has been shown to suppress proinflammatory cytokine expression as well and ameliorate heart damage by activating PPAR-*γ* and downregulating NF-*κ*B-mediated pathways [35]. Quercetin is also an important flavonoid component of MOE that may have contributed to its EAM ameliorating effects in our study, since it is shown that quercetin (20 mg/kg) may protect the heart from the damage in EAM conditions, via suppression of proinflammatory TNF-*α* and IL-17 and upregulation of anti-inflammatory cytokine IL-10 [14]. This study also used the *Dark Agouti* strain of rats, making it more comparable to our study. Other authors suggest that even in 10 mg/kg quercetin can protect the heart from EAM-induced damage via modulation of MAPK signaling cascade, more precisely by suppressing the myocardial endothelin-1 and also the mitogen-activated protein kinases (MAPK) [36]. Potent anti-inflammatory properties of quercetin have also been confirmed in other autoimmune diseases [37, 38]. Another flavonoid component of MOEs, catechin ,was also shown to exert protective effects in the EAM rat model by decreasing cardiac remodeling, inflammatory infiltrate, and fibrosis possibly via decreased expression of NF-*κ*B and ICAM-1 [27].

An important aspect in the pathophysiology of autoimmune myocarditis is the link between inflammation and excessive ROS production, otherwise oxidative stress, which we also evaluated in this study. Three weeks post-immunization, the EAM group was associated with a significant release of prooxidants O_2_^−^, H_2_O_2_, NO_2_^−^, and TBARS and impaired antioxidant defense system (decreased SOD, CAT, and GSH). This is consistent with earlier studies that reported various oxidative stress marker elevations in this disease, such as superoxide anion [36], lipid peroxidation products MDA and 4-hydroxynonenal, and TBARS [39, 40]. MOE treatment showed dose-dependent drop in the release of prooxidants O_2_^−^, TBARS, and NO_2_^−^, with strong antioxidant effect and free scavenging properties of this plant and its phenolic compounds being the most responsible for this effect. Various studies showed MOEs' ability to decrease oxidative stress in different cardiovascular models, even when using shorter time of exposition (7 or 14 days) than in this study [7, 33, 39]. We found that MOE treatment improves the systemic antioxidant status of EAM rats. The results of other studies support this finding, since there is evidence that MOEs can improve antioxidant capacity, mostly via SOD increment in models of LAD in vivo regional I/R injury and doxorubicin-induced cardiotoxicity. Additionally, *in vitro* investigations on MOEs confirmed its strong free radical scavenging properties on DPPH, ABTS, O_2_^−^, and NO_2_^−^ radicals, but also iron (II) chelating activity of that potentiates its antioxidant properties [41, 42]. Besides mentioned antioxidant effects of MOEs, it is important to emphasize that rosmarinic acid, dominant phenolic component of this plant, per se possesses strong effect in mitigating oxidative stress in different disorders [43]. The antioxidant power of rosmarinic acid is mainly based on its ability to stabilize membranes and stop free radical movement, thus preventing oxidation of the membranes [44].

Thus, oxidative stress ameliorating effect of the applied MOE treatment may also be one of the main mechanisms of autoimmune myocarditis improvement achieved by MOEs.

## 5. Conclusion

In the light of these findings, the present study suggests that ethanolic MOEs improve cardiac function and myocardial architecture and mitigate oxidative stress, thus preventing heart remodeling, DCM, and subsequent heart failure connected with human giant cell myocarditis and EAM. However, the most prominent reduction of cardiac inflammatory infiltration, fibrosis, and oxidative stress with preservation of ejection fraction has been observed with the highest dose of MOE, 200 mg/kg. This study is the first to provide evidence on *M. officinalis* effects in cardiac autoimmunity. However, additional experiments and future investigations are necessary and should help in revealing the exact mechanism of action of MOEs in EAM pathology. MOEs should be considered as a potentially helpful adjuvant therapy in patients with autoimmune myocarditis.

## Figures and Tables

**Table 1:** Effects of MOEs on body weight (Bw), heart weight (Hw), heart weight/body weight ratio (Hw/Bw ratio), spleen weight (Sw), and spleen weight/body weight ratio (Sw/Bw ratio). CTRL: control group; EAM: experimental autoimmune myocarditis group; MOE50, MOE100, and MOE200: groups of rats treated with either 50, 100, or 200 mg/kg of *Melissa officinalis* extract. Statistical significance at the level of *p* < 0.05^∗^compared to CTRL, ^#^compared to EAM, and ^¶^compared to MOE50. Data are expressed mean ± standard deviation.

	CTRL	EAM	MOE50	MOE100	MOE200
Bw (g)	215.29 ± 5.71	212 ± 14.97	195.88 ± 9.34	189 ± 10.06	194.25 ± 6.14
Hw (mg)	777.14 ± 34.50	1014.47 ± 110.89^∗^	937.50 ± 88.28^∗^	760.23 ± 67.17^#¶^	782.34 ± 29.73^#¶^
Sw (mg)	403.29 ± 23.61	447.57 ± 15.08^∗^	398.13 ± 23.90	351.38 ± 30.89^#^	335.12 ± 15.57^#¶^
Hw/Bw ratio (mg/g)	3.61 ± 0.24	4.81 ± 0.80^∗^	4.80 ± 0.60^∗^	4.02 ± 0.33^#¶^	4.10 ± 0.27^#¶^
Sw/Bw ratio (mg/g)	1.87 ± 0.08	2.12 ± 0.17^∗^	2.04 ± 0.20	1.86 ± 0.14^#^	1.71 ± 0.07^#¶^

**Table 2:** Effects of MOEs on systolic (SBP), diastolic blood pressure (DBP), and heart rate (HR). CTRL: control group; EAM: experimental autoimmune myocarditis group; MOE50, MOE100, and MOE200: groups of rats treated with either 50, 100, or 200 mg/kg of *Melissa officinalis* extract. Statistical significance at the level of *p* < 0.05^∗^compared to CTRL, ^#^compared to EAM, and ^¶^compared to MOE50. Data are expressed means ± standard deviation.

	CTRL	EAM	MOE50	MOE100	MOE200
SBP (mmHg)	133.60 ± 7.60	133.25 ± 6.13	124.75 ± 5.85	121.00 ± 5.72	113.80 ± 4.95^∗^^#¶^
DBP (mmHg)	81.40 ± 4.93	78.75 ± 6.08	79.25 ± 8.42	80.00 ± 5.35	68.60 ± 4.95
HR (beats/min)	367.60 ± 17.62	453.75 ± 40.54^∗^	373.50 ± 18.73^#^	333.00 ± 11.22^∗^^#¶^	332.60 ± 25.46^∗^^#¶^

**Table 3:** Effects of MOEs on echocardiographic parameters: interventricular septal wall thickness at end-systole and end-diastole (IVSs and IVSd), left ventricular internal diameter at end-systole and end-diastole (LVIDs and LVIDs), left ventricular posterior wall thickness at end-systole and end-diastole (LVPWs and LVPWd), fractional shortening (FS), and ejection fraction (EF). CTRL: control group; EAM: experimental autoimmune myocarditis group; MOE50, MOE100, and MOE200: groups of rats treated with either 50, 100, or 200 mg/kg of *Melissa officinalis* extract. Statistical significance at the level of *p* < 0.05^∗^compared to CTRL, ^#^compared to EAM, ^¶^compared to MOE50, and ^§^compared to MOE100. Data are expressed means ± standard deviation.

	CTRL	EAM	MOE50	MOE100	MOE200
IVSd (cm)	0.150 ± 0.039	0.174 ± 0.035	0.145 ± 0.032	0.148 ± 0.018	0.140 ± 0.012
LVIDd (cm)	0.452 ± 0.059	0.433 ± 0.027	0.450 ± 0.056	0.477 ± 0.046	0.516 ± 0.033
LVPWd (cm)	0.152 ± 0.027	0.193 ± 0.029^∗^	0.179 ± 0.015^#^	0.169 ± 0.024^#¶^	0.160 ± 0.007^#¶^
IVSs (cm)	0.175 ± 0.073	0.162 ± 0.020	0.141 ± 0.012	0.160 ± 0.033	0.154 ± 0.017
LVIDs (cm)	0.228 ± 0.041	0.338 ± 0.030	0.262 ± 0.034	0.301 ± 0.036	0.285 ± 0.046
LVPWs (cm)	0.160 ± 0.020	0.199 ± 0.047^∗^	0.179 ± 0.029^#^	0.171 ± 0.024^#¶^	0.161 ± 0.007^#¶^
FS (%)	49.19 ± 8.89	21.88 ± 4.55^∗^	41.59 ± 6.24^#^	38.92 ± 4.31^#^	44.96 ± 5.81^#¶§^
EF (%)	84.81 ± 7.74	50.33 ± 7.94^∗^	53.89 ± 4.85^∗^	72.47 ± 8.48^#¶^	81.44 ± 5.51^#¶§^

## Data Availability

The data used to support the findings of this study are available from the corresponding author upon request.
